# Single-layer biosensor for urinary prostate-cancer biomarkers through transition-metal-doped graphene: a DFT study

**DOI:** 10.1039/d5ra06085k

**Published:** 2025-11-04

**Authors:** Joshua Zhou, Xuan Luo

**Affiliations:** a National Graphene Research and Development Center Springfield Virginia 22151 USA

## Abstract

Early, non-invasive detection of prostate cancer (PCa) remains a major clinical challenge, as current screening methods carry significant drawbacks. Biosensors targeting urinary PCa biomarkers offer a promising alternative. Motivated by the recurrent appearance of sarcosine and furan-3-methanol in urinary volatilomics, and by the growing application of 2D nanomaterials in metabolite detection, we employed first-principles calculations to investigate pristine graphene and gold-, palladium-, and silver-doped graphenes as potential single-layer biosensors. We compared atomic optimizations, adsorption energies, band-gap shifts, charge-density differences, recovery times, conductivity changes, and theoretical sensing responses to identify the most effective sensor. Our results revealed that pristine graphene fails to adsorb either molecule; Au-doping binds sarcosine strongly but inadequately retains furan-3-methanol; Pd-doping leads to insufficient retention for both analytes; and Ag-doping enables rapid desorption of furan-3-methanol yet provides optimal sensing for sarcosine. Overall, Ag-doped graphene demonstrates strong potential as a room-temperature sensor for sarcosine, while detecting furan-3-methanol will require alternative chemistries or device architectures.

## Abstract 2

Ag-doped graphene is identified by DFT as the most responsive single-layer sensor for urinary sarcosine, combining strong adsorption, clear band-gap tuning, and viable room-temperature recovery.

## Graphical abstract

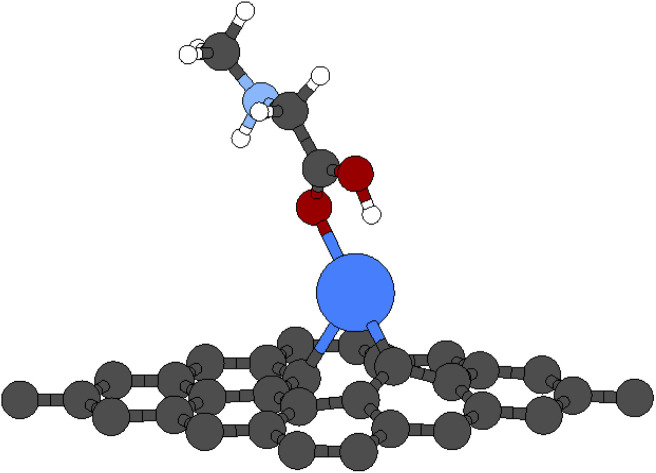

## 1. Introduction

Prostate cancer (PCa) is one of the most common malignancies in men, with over 1 400 000 new cases and around 375 000 new deaths worldwide in 2020.^1–6^ Once prostate cancer enters stage III, the five-year survival plummets from virtually 100% for organ-confined disease to 30.7% for distant metastases (stage IV), meaning roughly two-thirds of men die within five years once the tumour spreads.^7^ This high mortality rate thus makes early detection a critical preventative measure.^1,6–9^ However, current early-detection tools have important drawbacks.^10,11^ Digital rectal exams often miss cancer because many tumors do not change prostate texture,^12–14^ the previously revolutionary serum PSA is easily raised by benign enlargement or infection resulting in false positives, and even the standard transrectal ultrasound (TRUS) biopsy can miss up to half of true tumors.^10,11,15–18^ Other imaging, such as the multiparametric MRI or more recently the ^68^Ga-PSMA PET pinpoint suspicious areas more reliably, yet are expensive, time-consuming, and require expert readers.^10,11,19,20^ These limits have directed attention to the potential of metabolic signatures such as volatile organic compounds (VOCs) in easily obtained biofluids. Among the dozens of molecules observed, two rise to the top as early markers of tumour progression: the amino-acid derivative sarcosine (C_3_H_7_NO_2_), and the urinary volatile furan-3-methanol (C_5_H_6_O_2_).^21–26^

However, most current urine-based screens for prostate cancer metabolites still depend on multistep head-space GC-MS or related chemometric workflows that can take hours and call for dedicated analysts and hardware.^27,28^ Thus, research has begun to turn to alternative methods of detection. Currently, sarcosine has already inspired a fast-growing family of low-cost biosensors such as amperometric chips that read H_2_O_2_ released by sarcosine oxidase, organic-electro-chemical transistors dressed with Nafion, and mesoporous hybrids designed to suppress interference.^29–34^ In contrast, work on the urinary volatile furan-3-methanol is still confined almost entirely to discovery studies that flag it as a prostate-cancer marker in GC-MS headspace panels, with the most prominent sensor being an electronic-nose prototype utilizing broad sensor arrays rather than molecule-specific receptors.^24,26,35–37^ Together, these trends point to a clear opportunity. Both sarcosine, already validated across formats, and furan-3-methanol, still lacking a direct transducer, stand to benefit from research with two-dimensional (2D) nanomaterials whose effectiveness has already been proven through various studies.^23,28^

Amongst 2D nanomaterials, graphene, the one-atom-thick carbon monolayer that earned the 2010 Nobel Prize in Physics for its discoverers, is a benchmark material. The honeycomb lattice structure supports exceptionally mobile charge carriers, and its massive theoretical surface area provides abundant binding sites for analytes.^38–40^ Furthermore, graphene-family monolayers, doped or hybridised with noble-metal sites, have already proven they can translate femtomolar binding events into readable current shifts for a spectrum of VOCs, gases, and small metabolites, and extending this toolbox to the sarcosine and furan-3-methanol could be especially promising.^41^

Extensive theoretical and experimental research has mapped how graphene-based platforms interact with various small inorganic analytes.^23,28,41–46^ Pristine graphene shows measurable charge transfer mainly for strong electron-withdrawing gases such as NO_2_.^41,44,47^ Doping the lattice with transition metals (TM), especially Au, Pd, and Ag^46–53^ often magnifies this sensitivity. Palladium (Pd) substitution, for example, has been shown to adsorb SO_2_ particularly strongly.^46^ Another study by Ma *et al.*^48^ reveals the ability of Pd to drastically raise the conductivity of the monolayer for adsorption of CO, NH_3_, O_2_, and NO_2_. Comparable computational screens have examined gold (Au) and silver (Ag), revealing improvements in adsorption energy, charge redistribution, and sensitivity for pollutants from H_2_S to Cl_2_.^45,47,49,50^ Yet no study, computational or experimental, has quantified the interaction of TM-doped graphene with sarcosine or furan-3-methanol. Filling this gap is essential because these metabolites represent early, non-invasive markers of PCa progression, and clarifying their adsorption thermodynamics and electronic signatures on doped graphene will determine whether a single-layer chemiresistor can meet the sensitivity, selectivity, and regeneration requirements of point-of-care screening. We therefore apply first-principle calculations based on density-functional theory (DFT)^54^ with the dispersion-corrected PBE functional^55^ to compare pristine graphene with its Au-, Ag-, and Pd-doped variants to determine their effectiveness in detecting sarcosine and furan-3-methanol.

## 2. Method

### 2.1. Computational details

The density functional theory (DFT)^54^ incorporated into the ABINIT package^56^ implements the generalized gradient approximation (GGA)^55^ exchange–correlation functionals with a Perdew–Burke–Ernzerhof (PBE) format. We further used the projected augmented wave (PAW) method^57^ to produce pseudopotentials using the AtomPAW code.^58^ Hydrogen (H), carbon (C), nitrogen (N), oxygen (O), silver (Ag), gold (Au) and palladium (Pd) employed valence electron configurations 1s^1^, [He] 2s^2^2p^2^, [He] 2s^2^2p^3^, [He] 2s^2^2p^4^, [Kr] 4d^10^5s^1^, [Xe] 4f^14^5d^10^6s^1^ and [Kr] 4d^10^, and radius cutoffs of 0.99 bohr, 1.51 bohr, 1.41 bohr, 1.20 bohr, 2.50 bohr, 2.50 bohr, and 2.50 bohr, respectively.

### 2.2. Convergence details

The self-consistent field (SCF) iterations for total energy calculations were stopped when the total energy difference was less than 1.00 × 10^−10^ Ha twice consecutively. The kinetic energy cutoff, Monkhorst–Pack^59^*k*-point grids, and the vacuum height were considered converged when the difference in total energy was smaller than 0.0001 Ha (or 0.003 eV). Structural optimization was performed using the Broyden–Fletcher–Goldfarb–Shanno^53^ minimization with a maximum force tolerance of 2.00 × 10^−4^ Ha bohr^−1^ (or 0.01 eV Å^−1^).

### 2.3. Atomic structures

**Fig. 1:**
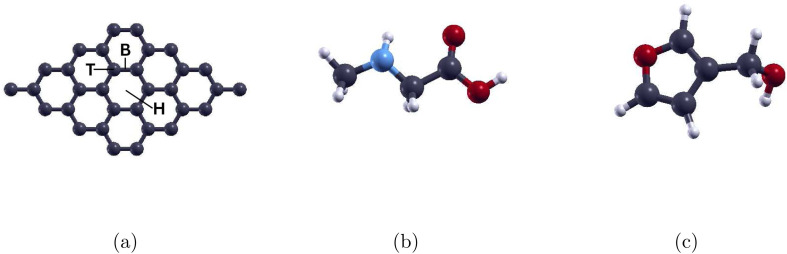
(a) Graphene supercell with hollow (H), bridge (B), and top (T) sites marked, (b) atomic structure of sarcosine, and (c) atomic structure of furan-3-methanol. Gray, white, red, and light blue colors are used to represent C, H, O, and N atoms, respectively.

We first performed atomic structure optimization calculations for the sarcosine and furan-3-methanol molecules, see Fig. 1(b) and (c). The 4 × 4 graphene supercell was then chosen for the adsorption of the two molecules. Due to the lack of pre-existing studies done on the adsorption of either molecule onto graphene, we studied the adsorption of the molecules on each of the hollow (H), bridge (B), and top (T) sites to find the most stable configuration. The three sites, as well as the graphene supercell consisting of 32 C atoms, are shown in Fig. 1(a). The Au-, Ag, and Pd-doped graphene monolayers were constructed through the substitution of one C atom with the corresponding TM atom.

### 2.4. Adsorption calculations

After each monolayer and molecule was prepared, the sarcosine and furan-3-methanol molecules were placed above the four graphene monolayers to create the studied molecule-monolayer systems. For pristine graphene, the molecules were placed above the monolayer on each of the considered adsorption sites. Once we relaxed the atomic structures of each system, we performed total energy calculations for: the molecule-adsorbed system, the pristine or TM-doped graphene monolayer, and the two molecules. The adsorption energy (*E*_ad_)^46^ for these systems were calculated using

1 *E*_ad_ = *E*_mol+monolayer_ − *E*_mol_ − *E*_monolayer_

where *E*_mol+monolayer_, *E*_mol_, and *E*_monolayer_ represent the total energies of the molecule-adsorbed system, pristine or TM-doped graphene, and the two molecules, respectively.

### 2.5. Electronic structure

Band structures were calculated for each system before and after the adsorption of the molecules. The band structures were then plotted using the high symmetry *k*-points *Γ*(0, 0, 0), *K*(2/3, 1/3, 0), *M*(1/2, 0, 0), and *Γ*(0, 0, 0). These points refer to the high symmetry points of the hexagonal Brillouin zone, a primitive cell within the reciprocal space of the structure, expressed in reciprocal lattice coordinates. We next observed the charge transfer^45^ to further understand the adsorption of the molecules onto the monolayers using the equation

2 Δ*ρ* = *ρ*_mol/monolayer_ − *ρ*_monolayer_ − *ρ*_mol_

where Δ*ρ* represents the net charge transfer, and *ρ*_mol/monolayer_, *ρ*_monolayer_, and *ρ*_mol_ represent the charge density of the molecule-monolayer system, the graphene monolayer, and the molecules, respectively. We then conducted calculations of the projected density of states (PDOS) on the adsorptions of the molecules onto the respective monolayers using the tetrahedron method. The atoms chosen for projection are atoms closest to the site of adsorption.

### 2.6. Sensor-performance metrics

To evaluate the viability of each monolayer as a potential biosensor for the studied biomarkers, we performed three primary calculations: evaluating the recovery time, conductivity, and sensitivity of the molecule-adsorbed systems.

To determine the reusability of each sensor, we calculated the minimum time for desorption from each monolayer, where the recovery time (*τ*)^53^ was calculated as

3 *τ* = *v*^−1^ e^−*E*_ad_/*k*_B_*T*^

where *v* represents the attempt frequency (*v* = 10^12^ s^−1^ for visible light), *k*_B_ represents the Boltzmann constant (*k*_B_ = 8.617 × 10^−5^ eV K^−1^), and *T* represents temperature (*T* = 300 K for room temperature).

The conductivity (*σ*)^53^ of the systems was then calculated using the equation4
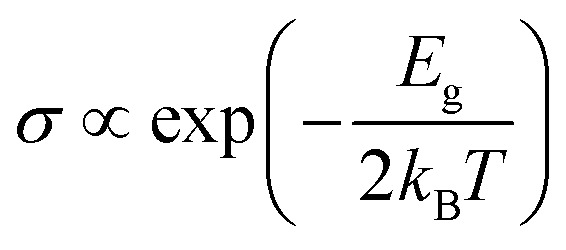
where *E*_g_, *k*_B_, and *T* represent the band gap of the systems, the Boltzmann constant, and the temperature, respectively.

The final metric we examined was the sensing response (*S*),^53^ or the variation in electrical resistance before and after the adsorption of the molecules. The equation used was defined as5
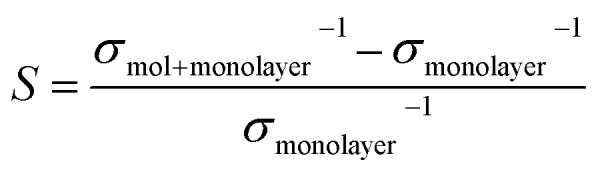
where *σ*_mol+monolayer_ and *σ*_monolayer_ represent the conductivity of the molecule-monolayer system and the monolayer, respectively.

## 3. Results and discussion

We initially studied the adsorption properties of the molecules onto pristine graphene (Gr). Then, to improve the sensitivity of graphene towards said molecules, TM atoms were substitutionally doped into the monolayer. Finally, the TM-doped graphene systems were analyzed before and after molecular adsorption. For each system, we studied and compared the atomic structure, adsorption energy, recovery time, band structure, charge transfer, sensitivity, and conductivity to find the most effective sensor for each molecule.

### 3.1. Molecules on pristine graphene

For the free sarcosine molecule, the calculated C–O bond length is 1.21 Å, and the bond angles of H–N–H and O–C–O are 113.68° and 123.18°, respectively. For the free furan-3-methanol molecule, the calculated C–O bond length is 1.36 Å, while the O–C–O bond angle is 106.75°. The fully relaxed atomic structure of Gr converged to a lattice constant of 2.47 Å, listed in Table 1 alongside the optimized parameters of each studied monolayer, which is consistent with many previous studies, such as Yang's 2018 study^60^ that reported a lattice constant of 2.46 Å. As expected for Gr based on existing research,^39,40,60^ the electronic dispersion retained a zero direct band gap.

**Table 1:** Optimized lattice constant *a* (Å), bond length *d* (Å) between dopant and monolayer, and electronic band gap *E*_g_ (eV) for pure 4 × 4-graphene monolayers (Gr), Au-doped graphene monolayer (Au-Gr), Pd-doped graphene monolayer (Pd-Gr), and Ag-doped graphene monolayer (Ag-Gr)

	Gr	Au-Gr	Pd-Gr	Ag-Gr
*a* (Å)	2.47	2.50	2.50	2.54
*d* (Å)	N/A	2.08	1.94	2.21
*E*_g_ (eV)	0.000	0.054	0.295	0.187

**Fig. 2:**
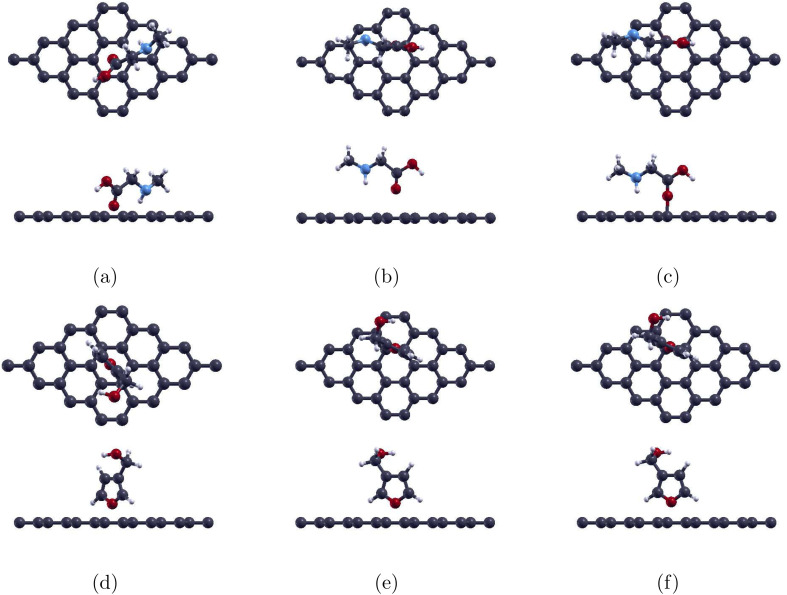
Top and side views of the atomic structures of sarcosine and furan-3-methanol on the (a) and (d) hexagonal, (b) and (e) bridge, and (c) and (f) top adsorption sites of pure graphene prior to relaxation. Gray, white, red, and light blue colors are used to symbolize C, H, O, and N atoms, correspondingly.

After the optimization of the atomic structures of Gr, sarcosine, and furan-3-methanol, we observed the adsorption of the two molecules onto the monolayer over the three archetypal adsorption sites. The unrelaxed starting configurations of these six systems can be seen in Fig. 2. Although we accounted for van der Waals forces, the systems were unable to relax to satisfactory states. This reveals that neither molecule forms a stable bond with Gr. Gr was therefore judged unsuitable as a stand-alone sensing platform for sarcosine or furan-3-methanol, motivating the exploration of TM doping. This unsuitability further informed our decision to not perform further tests on Gr such as calculating the band structures.

### 3.2. Molecules on Au-doped graphene

**Fig. 3:**
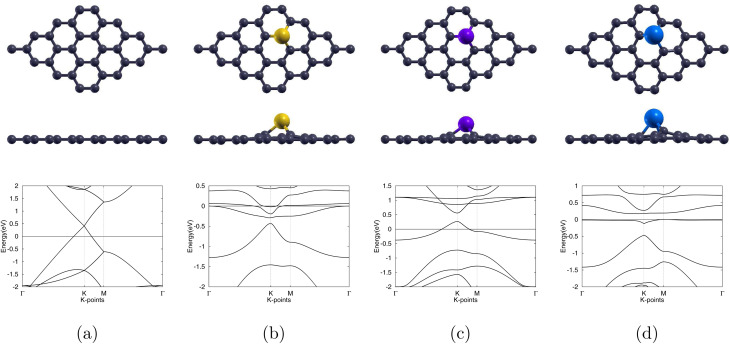
Optimized atomic structures (top and side views) together with the corresponding electronic band structures of (a) pristine graphene, (b) Au-doped graphene, (c) Pd-doped graphene, and (d) Ag-doped graphene monolayers. Gray, yellow, purple, and blue colors are used to symbolize C, Au, Pd, and Ag atoms, correspondingly.

Gold (Au) was selected as the first dopant owing to its widely reported ability to enhance Gr's reactivity. We substituted a C atom of the Gr monolayer with the dopant, Au, to create the 4 × 4 Au-doped graphene (Au-Gr) monolayer, as previous studies have shown that substitutional doping of Gr has the most promising results.^51–53^ Structural optimisation shows the Au centre protruding from the plane and distorting the adjacent six-membered ring (6MR). The dopant also opens the zero graphene band gap to a 0.054 eV band gap. This optimized structure alongside the band structure are shown in Fig. 3(b).

**Fig. 4:**
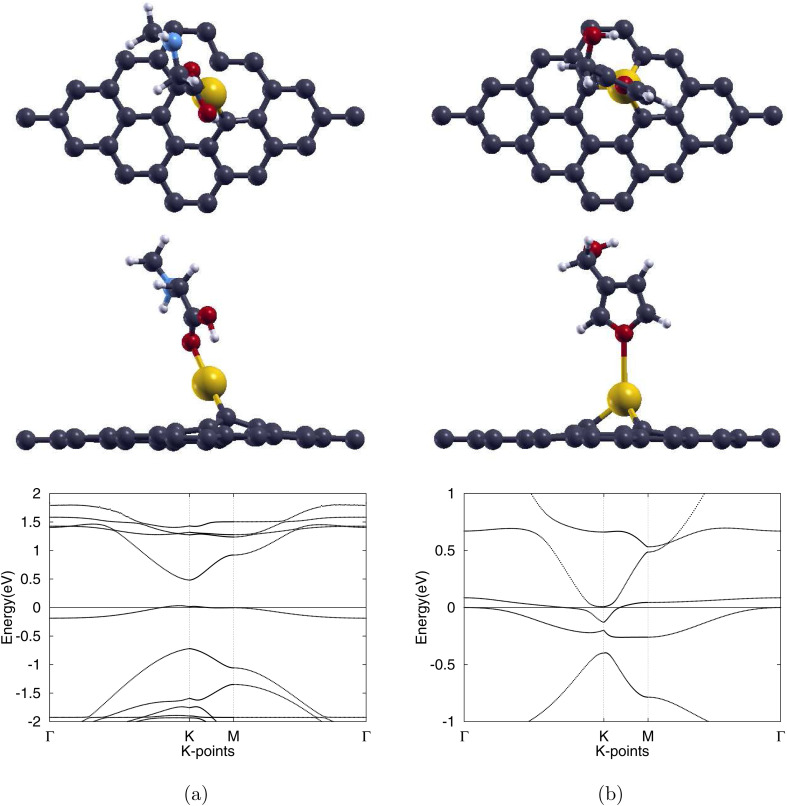
Optimized atomic structures (top and side views) together with the corresponding electronic band structures of (a) sarcosine adsorbed on Au-doped graphene and (b) furan-3-methanol adsorbed on Au-doped graphene. Gray, white, red, light blue, and yellow colors are used to symbolize C, H, O, N, and Au atoms, correspondingly.

After optimizing Au-Gr, we then investigated the adsorption of sarcosine and furan-3-methanol onto the monolayer. When sarcosine or furan-3-methanol is placed with its most reactive O atom directly above the Au site and the structure, see Fig. 4, is relaxed, creating the Sar/Au-Gr and 3f/Au-Gr systems, contrasting bonding responses emerge. Sarcosine reduces the three Au–C links to a single bond shortened to 2.01 Å, which is 3.3% shorter than the original bond length. On the other hand, furan-3-methanol preserves all three bonds, slightly elongating them by 0.5% to 2.09 Å. As illustrated in Table 2, final Au–O distances measure 2.18 Å for sarcosine and 2.87 Å for furan-3-methanol.

**Table 2:** Adsorption energy *E*_ad_ (eV), final adsorption distance *h* (Å) between active O atom of molecules sarcosine or furan-3-methanol and TM atom of graphene, band gap *E*_g_ (eV), and recovery time *τ* (s) for two molecules adsorbed on graphene

System	*E*_ad_ (eV)	*h* (Å)	*E*_*g*_ (eV)	*τ* (s)
Sar/Au-Gr	−0.934	2.18	0.449	4.88 × 10^3^
3f/Au-Gr	−0.169	2.87	Metallic	7.02 × 10^−10^
Sar/Pd-Gr	−0.379	2.31	0.318	2.30 × 10^−6^
3f/Pd-Gr	−0.181	2.79	0.340	1.10 × 10^−9^
Sar/Ag-Gr	−0.769	2.21	0.376	8.21
3f/Ag-Gr	−0.449	2.31	0.337	3.56 × 10^−5^

The corresponding adsorption energies of −0.934 eV for Sar/Au-Gr and −0.169 eV for 3f/Au-Gr translate, *via*eqn (3), into recovery times of 4.88 × 10^3^ s and 7.02 × 10^−10^ s, respectively. This indicates that Sar/Au-Gr has a prohibitively long recovery time for a regenerable chemiresistor, whereas 3f/Au-Gr has an effectively instantaneous recovery on experimental timescales, leaving little room for observation. Thus, Au-Gr is deemed an unsuitable biosensor for the studied analytes, motivating us to study palladium as an alternative dopant.

### 3.3. Molecules on Pd-doped graphene

Palladium doping (Pd-Gr) follows the same substitutional protocol. The relaxed monolayer visible in Fig. 3(c) shows the Pd atom lifted above the lattice and bonded to neighbouring carbons by three 1.94 Å bonds, matching the 1.96 Å benchmark of Ma *et al.*^48^ The lattice constant of the central graphene 6 MR of the Pd-Gr cell expanded to 2.50 Å. The band structure of Pd-Gr, see Fig. 3(c), shows the expansion of the graphene band gap to 0.295 eV.

**Fig. 5:**
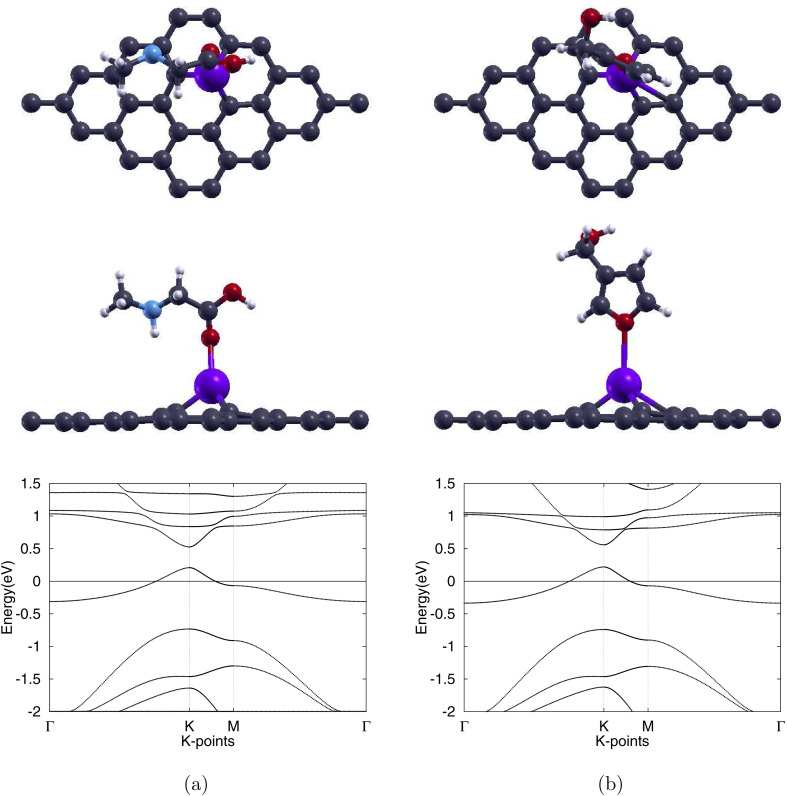
Optimized atomic structures (top and side views) together with the corresponding electronic band structures of (a) sarcosine adsorbed on Pd-doped graphene and (b) furan-3-methanol adsorbed on Pd-doped graphene. Gray, white, red, light blue, and purple colors are used to symbolize C, H, O, N, and Pd atoms, correspondingly.

**Fig. 6:**
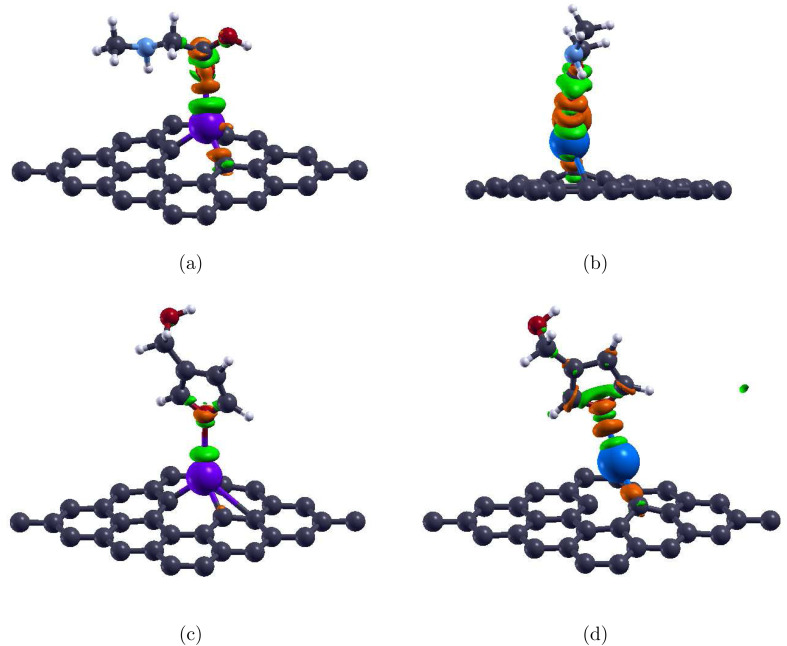
Charge density of the monolayer-molecule systems with an isovalue of 0.014 e Å^−3^: sarcosine adsorbed on (a) Pd-doped graphene and (b) Ag-doped graphene; and furan-3-methanol adsorbed on (c) Pd-doped graphene, and (d) Ag-doped graphene. Gray, white, red, light blue, yellow, purple, and blue colors are used to symbolize C, H, O, N, Au, Pd, and Ag atoms, respectively. The orange regions indicate the electron acceptor, while the green regions indicate the electron donor.

After adsorption of sarcosine to create the Sar/Pd-Gr system, the three Pd–C bonds lengthen only slightly to 1.96 Å, an increase of 1.0%. With the adsorption of furan-3-methanol, creating the 3f/Pd-Gr system, the bonds lengthen even less to 1.95 Å, an increase of 0.5%. All three bonds remain intact under the adsorption of either molecule. These relaxed structures are illustrated in Fig. 5. The optimized Pd–O distances shown in Table 2 are 2.31 Å for Sar/Pd-Gr and 2.79 Å for 3f/Pd-Gr. Calculated adsorption energies of −0.379 eV for Sar/Pd-Gr and −0.181 eV for 3f/Pd-Gr lead to recovery times of 2.30 × 10^−6^ s and 1.10 × 10^−9^ s, respectively. These values, however, still fall outside the general recovery time range for like sensors. This is further supported through the illustration of the charge transfer isosurface in Fig. 6(a and b) with green and orange areas representing areas of charge depletion and accumulation, respectively. The visual lack of a significant overlapping area indicates a negligible exchange of electrons between the O atom of analytes and the Pd atom, leading us to conclude that Pd-Gr, too, is unsuitable for the detection of either sarcosine or furan-3-methanol. We were then, due to the unpromising results shown for Pd-Gr and Au-Gr, prompted to select a final dopant, silver.

### 3.4. Molecules on Ag-doped graphene

**Fig. 7:**
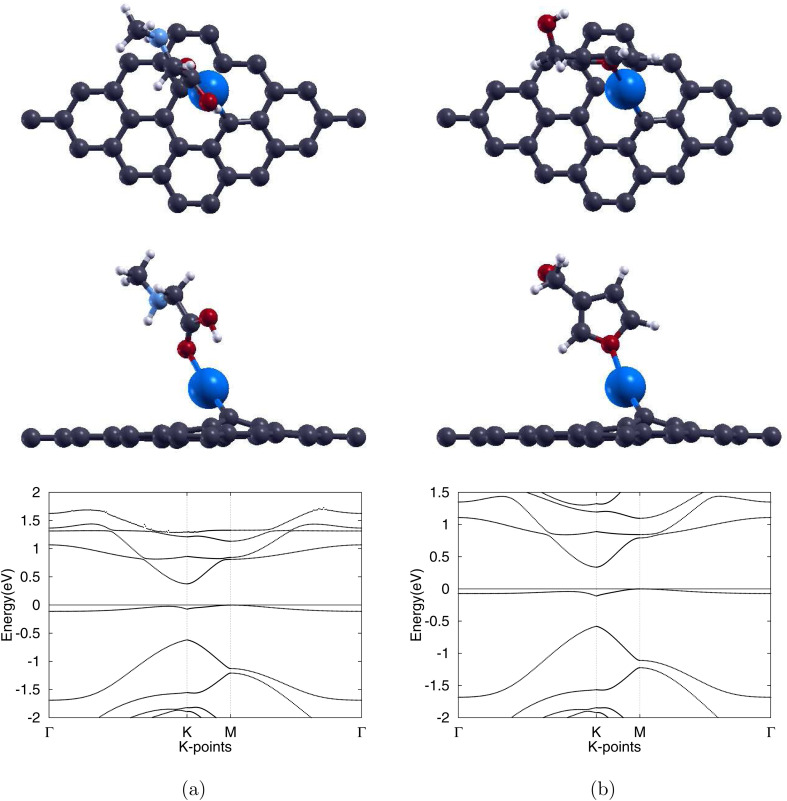
Optimized atomic structures (top and side views) together with the corresponding electronic band structures of (a) sarcosine adsorbed on Ag-doped graphene and (b) furan-3-methanol adsorbed on Ag-doped graphene. Gray, white, red, light blue, and blue colors are used to symbolize C, H, O, N, and Ag atoms, correspondingly.

Silver substitution (Ag-Gr) yields a pronounced out-of-plane Ag centre, see Fig. 3(d), with the three 2.25 Å Ag–C bonds in line with earlier studies.^52^ The lattice expands to 2.54 Å and the graphene band gap opens to 0.187 eV, with the band structure of Ag-Gr shown in Fig. 3(d). Upon molecular adsorption, with the fully atomically optimized structures displayed in Fig. 7, each system is left with a single Ag–C bond with lengths of 2.09 Å for the sarcosine adsorbed Sar/Ag-Gr system, a decrease of 7.1%, and 2.10 Å for the furan-3-methanol adsorbed 3f/Ag-Gr system, a decrease of 6.7%. The final Ag–O distances converge to 2.21 Å for Sar/Ag-Gr and 2.31 Å for 3f/Ag-Gr.

Adsorption energies of −0.769 eV for Sar/Ag-Gr and −0.449 eV for 3f/Ag-Gr yield recovery times of 8.21 s and 3.56 × 10^−5^ s, respectively. Although the recovery time for the desorption of sarcosine lies within the expected experimental range, the recovery time for 3f/Ag-Gr is insufficient for any practical observation or real-time analysis. Thus, the following analysis focuses only on the potential of Ag-Gr as a sensor for sarcosine.

To further observe the adsorption properties of sarcosine, we calculated the charge transfer for the adsorbed Ag-Gr system. As illustrated by the isosurface in Fig. 6(b), the electrons deplete near the Ag dopant while accumulating near the O atom of the molecules. Furthermore, the large overlapping area indicates a significant exchange of electrons between the molecule and the monolayer.

**Fig. 8:**
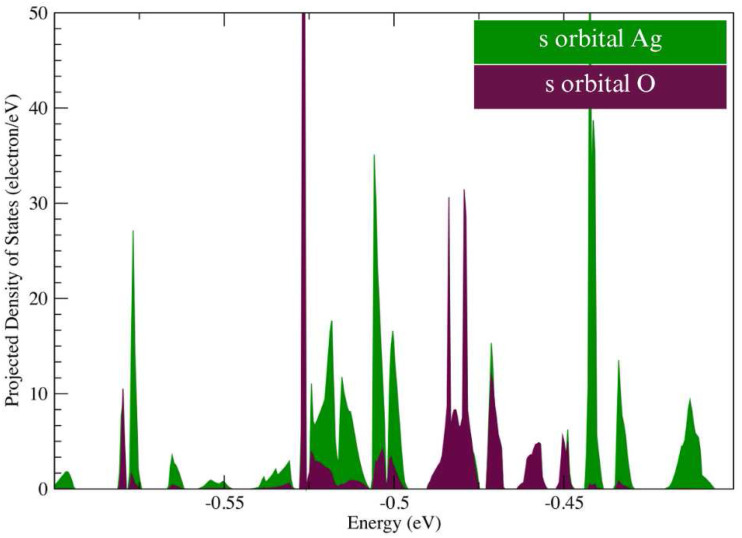
PDOS of Ag-doped graphene and sarcosine on Ag-doped graphene.

The post-adsorption band gap opens up to 0.376 eV, significantly above the pre-adsorption 0.187 eV value. Finally, we plotted the projected density of states for the adsorption. This is shown in Fig. 8, plotting the s orbitals of the Ag atom of Ag-Gr and the bonded O atom of sarcosine. There is overlap in the PDOS of the interacting atoms from −0. 5 eV to −0.3 eV, supporting our previous conclusions about the interaction between sarcosine and Ag-doped graphene. This positions Ag-Gr as the most promising biosensor among the series and its −0.769 eV adsorption energy categorizes the interaction as chemisorptive.^61^

However, the selectivity of Ag-Gr towards sarcosine in a practical sensor is a crucial issue. Urine is an extremely intricate biofluid containing various metabolites that could likely interfere with the adsorption of sarcosine on Ag-Gr. In general, 2D nanomaterial sensors can suffer selectivity issues in complex biofluids with many chemically similar metabolites. Differences in various properties such as adsorption energy, band gap, final atomic distance, and density of state could theoretically aid in the differentiation between molecules. However, given the hundreds of metabolites present within urine, it is not feasible within the scope of this paper to analyze metrics for each possible interferent. Because of this, we propose an upstream cavitand-based capture module to pre-process urine, using the tetraphosphonate cavitand's selective filtering of sarcosine.^62^ The Ag-Gr sensor would then be responsible for detecting the filtered sarcosine after the suggested preprocessing, creating a combined system for the detection of sarcosine. We have not considered an additional method for filtering furan-3-methanol because we do not believe that our research proves the potential of 2D-nanomaterials in detecting furan-3-methanol.

### 3.5. Comparative analysis

The collective trends in adsorption energy, recovery time, charge transfer, and band gap shifts confirm that TM doping endows graphene with genuine sensing capability toward sarcosine; however, this conclusion can not be made for furan-3-methanol. Au- and Pd-Gr monolayers are both unsuitable for the detection of either analyte. For the detection of furan-3-methanol, Ag-Gr holds the same conclusion; however, the results shown for sarcosine are far more promising. The doped monolayer thereby emerges as the most promising platform for sensitive detection of sarcosine under ambient conditions.

## 4. Conclusion

First-principles calculations based on DFT were used to examine, in parallel, pristine graphene and its gold-, palladium-, and silver-doped derivatives as single-layer sensors for the prostate-cancer biomarkers sarcosine and furan-3-methanol. Pristine graphene was confirmed to interact only weakly with either molecule, providing neither sustained adsorption nor measurable electronic response. Gold substitution immobilised sarcosine and provided an insufficient retention time for furan-3-methanol. Both outcomes are incompatible with real-time sensing. Palladium doping likewise resulted in recovery times that are inadequate for observing either molecule. While silver provided a similarly short recovery time for furan-3-methanol, it provided the most consistent results, achieving chemisorption powerful enough to hold the analytes yet labile enough to allow regeneration, for sarcosine. Furthermore, the silver-doped monolayer produced the most pronounced shift in the graphene band gap and, by extension, the largest predicted conductivity response. Conclusively, these qualitative trends identify silver-doped graphene as the most promising single-layer candidate for ambient-temperature detection of sarcosine. Regrettably, our calculations were unable to determine a viable biosensor for furan-3-methanol, a gap for future studies.

The present study, though comprehensive at the first-principles level, is not free from uncertainty. Density-functional theory inherits the usual functional-choice and basis-set limitations; finite super-cell sizes can under- or overestimate adsorption energy, and the theoretical nature of our experiment leaves open the question of how the predicted responses will translate to real biofluids and practical transducers. Human error in constructing initial geometries or interpreting convergence criteria can also introduce bias. Future work should therefore include larger and more defect-rich cells, and, critically, experimental validation of our results under realistic operating conditions. Extending the dopant palette, exploring co-doping strategies, testing different monolayer bases, and integrating the most promising monolayer into field-effect or chemiresistive architectures will help determine whether the computationally identified advantages of silver can be realized in real-time biosensors for early prostate cancer screening.

## Conflicts of interest

There are no conflicts to declare.

## Data Availability

Images for this article, including .eps files for charge densities, atomic structures, and band structures, are available at Zenodo under the name “Single-Layer Sensor, Prostate Cancer, DFT, Joshua Zhou, IMAGES” at https://doi.org/10.5281/zenodo.16891329. Data for this article, including ABINIT input and output files, are available at Zenodo under the name “Single-Layer Sensor, Prostate Cancer, DFT, Joshua Zhou, DATA” at https://doi.org/10.5281/zenodo.16891353.
